# 3D-Printed Dual Photo- and Thermally Responsive Materials for Smart Adaptability

**DOI:** 10.1021/acs.macromol.5c00567

**Published:** 2025-07-15

**Authors:** Tao Zhang, Gianni Pacella, Kunlin Chen, Ting Ye, Giuseppe Portale, Vincent S. D. Voet, Rudy Folkersma, Katja Loos

**Affiliations:** † Macromolecular Chemistry and New Polymeric Materials, Zernike Institute for Advanced Materials, University of Groningen, Nijenborgh 3, 9747 AG Groningen, The Netherlands; ‡ Circular Plastics, 84808 Academy Tech & Design, NHL Stenden University of Applied Sciences, Van Schaikweg 94, 7811KL Emmen, The Netherlands; § Key Laboratory of Eco-Textile, Ministry of Education, School of Textile Science and Engineering, 66374 Jiangnan University, Wuxi 214122, China

## Abstract

The development of intelligent 3D printing materials with photo- and thermally responsive properties remains a challenge, particularly for sustainable applications requiring reprocessability, multifunctionality, and precise control over dynamic behaviors. Herein, we report a solvent-free photoprintable ink featuring dual dynamic covalent bonds (DCBs), designed for photo- and thermally responsive 3D printing. The resin combines dynamic disulfides and β-hydroxy esters, further enhanced with photoswitchable spiropyran additives, enabling tunable photochromic and thermochromic properties. This material also exhibits shape memory functionality, allowing simultaneous shape and color transitions under heat. Printed prototypes demonstrate exceptional thermal stability and multifunctionality, supporting applications in anticounterfeiting, imaging, and sensing. These results provide a sustainable and innovative solution for intelligent 3D printing materials, bridging the gap between multifunctionality and reprocessability. By advancing the capabilities of responsive materials, this work paves the way for transformative progress in adaptive manufacturing and practical applications in next-generation functional devices.

## Graphical abstract

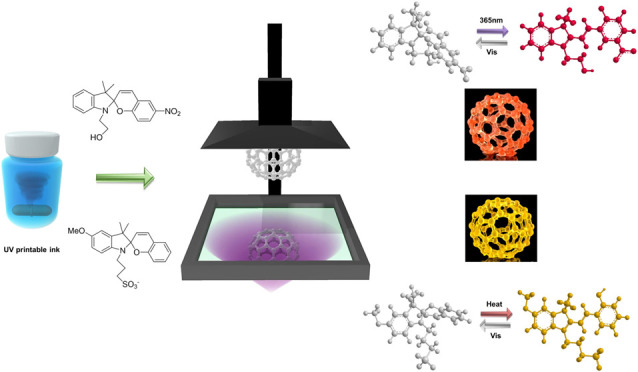

## Introduction

Masked stereolithography (mSLA) is a resin-based 3D printing technology analogous to digital light processing (DLP). While both techniques use photopolymerization to selectively cure liquid resin layer by layer, they differ in the light modulation mechanism. DLP utilizes a digital micromirror device to project patterned light onto the resin surface, with each micromirror representing a voxel in the print. In contrast, mSLA employs an LCD screen as a dynamic mask placed between a UV light source and the resin vat. The LCD selectively blocks or transmits light to cure only the desired cross-section of each layer. Due to the affordability of LCD screens, mSLA become widely used technique for desktop-scale additive manufacturing. It is a versatile 3D printing technology that offers high precision, fast printing speeds, and smooth surface finishes due to its simultaneous layer exposure technique. These qualities make mSLA a suitable fabrication technology for electronics,^1^ sensors,^2^ tissue engineering,^3^ porous carriers,^3^ and clinical apparatuses.^4^ However, despite its wide range of application areas, issues with currently available printing materials, such as the requirement of extensive postprocessing, poor mechanical resistance (fragility) and high consumable cost, limit the potential use of mSLA. Furthermore, most of the materials used for mSLA are not reusable, which causes waste and pollution problems.^5–7^

To overcome the existing issues with SLA printing materials, incorporating dynamic covalent chemistry into the chemical composition provides feasible pathways for developing closed-loop recyclable inks,^5^ printed elastomers with variable mechanical properties,^8^ robust prints with ductile properties at elevated temperature,^9^ and shape-programmable processes. Traditional covalent networks are typically static and stable, making it difficult to break and reform. In contrast, dynamic covalent networks (DCNs), composed of cross-linked polymer structures in dynamic equilibrium via reversible covalent bonds, combine the high mechanical and thermal resistance of thermosets with the reshaping, reprocessing, shape reconfiguration, and memory capabilities of thermoplastics through reversible bond exchange.^10^ The polymer network’s topology is the key determinant of its shape memory and reprogramming abilities. Upon heating, the increased mobility of polymer chains allows for rearrangement under applied stress, enabling shape reconfiguration. Upon cooling, the stabilized network locks in the new shape. Moreover, the incorporation of dynamic covalent bonds (DCBs) enables reversible bond breakage and reformation at elevated temperatures, facilitating the reorganization of the polymer network’s topology. This dynamic cross-linking process allows the material’s internal structure to adjust under thermal stimuli. By employing these reversible bonds in UV-curable inks, shape-adaptive and thermally adaptable printable prototypes can be developed. The commonly used reversible chemistries include, disulfide bonds,^11^ transesterification reactions,^12^ Diels–Alder reactions,^13^ imines,^14^ carbamate,^8^ hindered urea,^15^ and boronate esters.^16^ In this work, the combination of disulfide bond exchange and β-hydroxy ester transformations, facilitated by a transesterification catalyst, allows for enhanced control over polymer chain rearrangement and topological reorganization at elevated temperatures.^17^ The use of these two chemistries provides complementary mechanisms: disulfide bond exchange offers rapid reversibility, while β-hydroxy ester transformations enable more gradual, tunable rearrangement, together improving the adaptability of the material’s structure.^18^ This capacity is essential for facilitating self-healing, shape reconfiguration, and memory effects. These properties are critical for developing materials with shape memory and configurability, making them highly valuable for additive manufacturing.^19^

In addition to incorporating dynamic building blocks or other versatile moieties into the polymeric network to enhance the fundamental physicochemical properties of printing materials, recent advancements in ink technology have driven significant interest in the development of multifunctional printing components. By simply mixing additives with ink, prints can be readily tailored to meet a wide range of functional requirements. Inks containing multifunctional additives demonstrate improved properties and functionalities, such as enhanced mechanical strength,^20^ thermal stability,^20^ electrical conductivity,^21^ and superior chemical,^7^ optical,^22^ and biological^23^ characteristics, thereby accelerating the advancement of the 3D printing field.

Molecular switches, which can reversibly change molecular conformation and color in response to external stimuli such as light, pH, and temperature, represent an exciting development in functional inks. These functional inks expand the range of applications and lead to the development of innovative printed parts with superior performance characteristics. The unique properties of photochromic and thermochromic inks make them highly versatile and appealing for various functions, including apparel, textiles, fluorescence imaging,^24^ smart lenses,^25^ security printing,^26^ packaging, indicators,^27^ hazard warnings, and smart windows for energy-efficient buildings.^25,28^ Among the molecular switches, spiropyrans are one of the earliest reported organic photochromic systems, yet highly interesting due to their multiresponsive behavior to light, heat, and pH. Spiropyrans are colorless, but UV excitation causes a ring-opening reaction associated with the breakage of the C–O bond, which transforms the molecule into its conjugated merocyanine (MC) form, that absorbs visible light (500–600 nm) and, hence, appears colored.^24^

Finally, traditional inks, which rely on solvent-based formulations, release volatile organic compounds (VOCs) and hazardous chemicals during production and postprocessing, contributing to environmental impacts and health risks.^9^ This underscores the need for more sustainable alternatives. The development of environmentally friendly printing inks is essential for aligning industrial practices with sustainability goals.^29^

In this work, we address critical limitations in materials for mSLA 3D printing by developing an affordable photoprintable ink that incorporates dual dynamic covalent bonds (disulfides and β-hydroxy esters) via a straightforward, solvent-free process. The cross-linker (DPBMA) was synthesized to introduce dual dynamic disulfides and β-hydroxy esters into its main chain. Successful synthesis was achieved through a straightforward preparation method, where glycidyl methacrylate (GMA) was esterified with 3,3′-dithiodipropionic acid at a 2:1 molar ratio at 100 °C, using triazabicyclodecene (TBD) as a transesterification catalyst (Figure 1a). Its chemical structure was confirmed with ATR-FTIR spectroscopy (Figure S2) and ^1^H NMR (Figure S3). The appearance of absorbance at 3460 and 1636 cm^–1^, along with the absence of intensity at 1689 and 910 cm^–1^, indicates the successful grafting of methacrylate while maintaining the unsaturated moiety (Figure S2). Additionally, the protons at 6.0 and 5.7 ppm and the signals from the f, h, and g positions further support this finding (Figure S3). For the photoprinting (Figure 1b), 2-hydroxyethyl methacrylate (HEMA, 20% w/w), a monofunctional diluent, was mixed with DPBMA (80% w/w) and a photoinitiator (BAPO, 0.5% w/w). Spiropyran molecular switches capable of photochromic responses (SP1, reddening upon exposure to 365 nm UV lightand, and SP2, yellowing upon 50 °C or above) were mixed into the liquid photosensitive resin, resulting in a SP1 containing resin and a SP2 containing resin, to investigate its responsive behavior (Figure 1c). This innovative approach minimizes the need for extensive postprocessing while enhancing mechanical strength and durability, effectively overcoming challenges related to material fragility and enabling tunable shape adjustments. Moreover, the dual dynamic bonds confer reusability and reprocessability, directly addressing environmental concerns such as waste and pollution associated with conventional thermoset materials. By integrating two spiropyran additives with distinct photochromic behaviors, we developed self-healable resins capable of color switching upon irradiation or heating/cooling stimuli. For instance, a printed flower demonstrates a dual-response mechanism: it opens and transitions from colorless to yellow under heat, showcasing the potential for developing and enriching interactive 4D printing materials. Furthermore, our strategy offers a rapid stress-relaxation polymer network, solving problems related to high consumable costs and offering a sustainable, cost-effective alternative. These advancements collectively demonstrate a substantial improvement in the material properties for mSLA printing, unlocking new opportunities for versatile, environmentally friendly, and high-performance applications.

**Figure 1:**
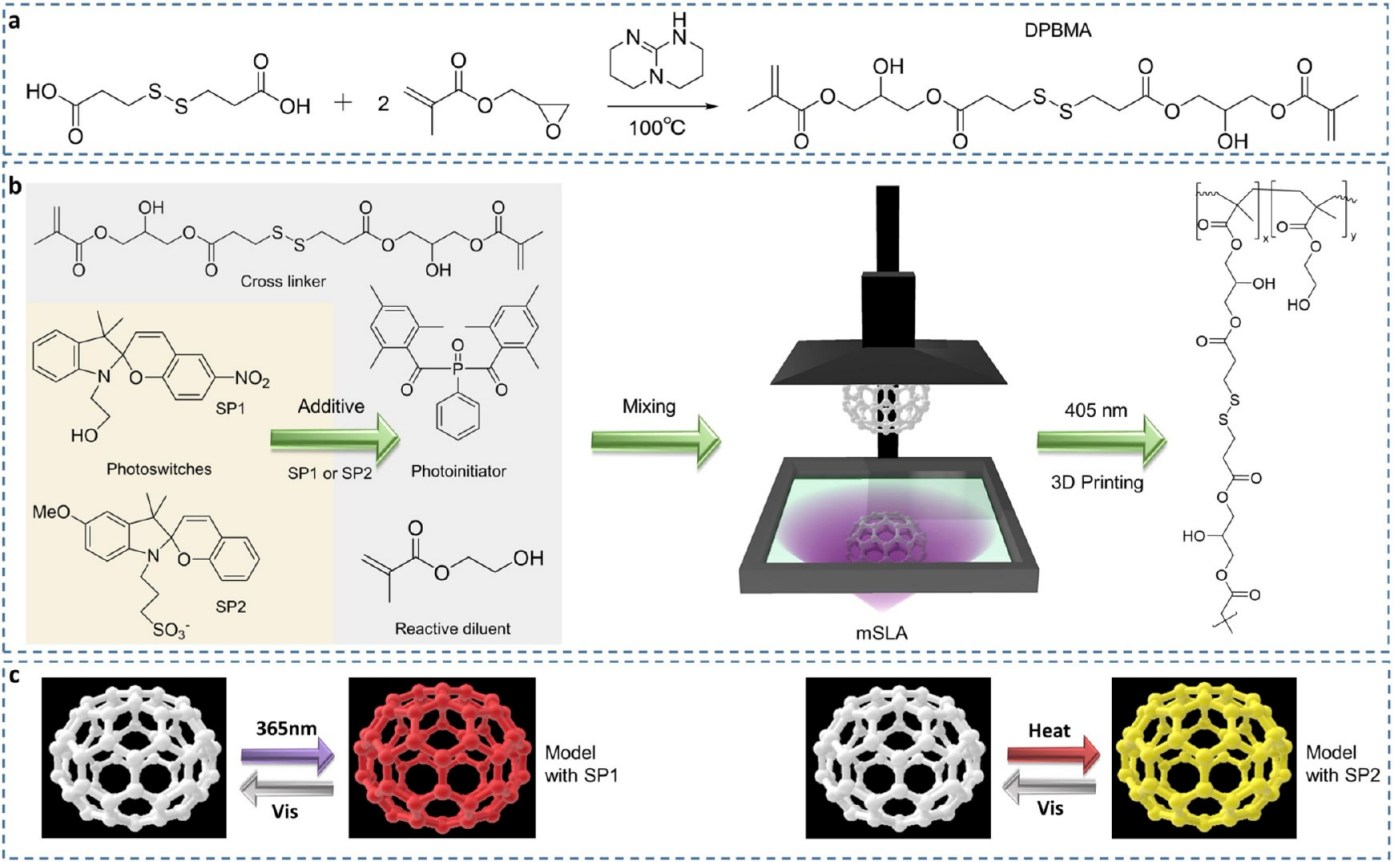
3D printing of dynamic chemistry precursors and photoswitches additives. (a) Synthesis route of the DPBMA cross-linker via one-step preparation from 3,3′-dithiodipropionic acid and glycidyl methacrylate at 100 °C for 2 h, using TBD as the catalyst. (b) Chemical composition of 3D printing ink, consisting of a cross-linker DPBMA, photoinitiator BAPO, photoswitches SP1 or SP2, and reactive diluent (HEMA). (c) Color changes triggered by external stimuli (e.g., 365 nm UV light, heat, visible light) are enabled by incorporating photoreswitch additives, such as SP1 or SP2, into the prints. SP1 facilitates UV-induced transitions, while SP2 provides responsiveness to heat.

## Methods

### Materials

2-Hydroxyethyl methacrylate (HEMA, 99%, containing ≤ 50 ppm monomethyl ether hydroquinone as an inhibitor), 2-propanol (99.5%), and dimethyl sulfoxide-d6 ((CD3)_2_S=O, 99.5 atom % D) were purchased from Sigma–Aldrich. Phenylbis­(2,4,6-trimethyl benzoyl) phosphine oxide (BAPO, 97%), glycidyl methacrylate (>95%, stabilized with MEHQ), and 3,3′-dithiodipropionic acid (>99.0%) were purchased from TCI (Tokyo Chemical Industry, Japan). Triazabicyclodecene (TBD, 97%), 2-(3′,3′-dimethyl-6-nitrospiro­[chromene-2,2′-indolin]-1′-yl)­ethanol (SP1, 93%) were acquired from BLD Pharmatech GmbH. All chemicals were used as received. Merocyanine photoacid (SP2) molecules were prepared according to a reported procedure.^30,31^ The ^1^H NMR spectrum corresponding to the chemical structure is presented in Figure S1.

### Preparation of the Ink Precursors

(3,3′-Disulfanediylbis­(propanoyl))­bis­(oxy)­bis­(2-hydroxypropane-3,1-diyl) bis­(2-methyl acrylate) (DPBMA) was synthesized through straightforward polymerization as follows. First, glycidyl methacrylate (28.4 g, 190 mmol), 3,3′-dithiodipropionic acid (20.2 g, 95 mmol), and TBD (0.5% w/w, 0.243 g) were added to a conical flask. The mixture was stirred using a magnetic stirrer at a speed of 200 rpm, while the heating plate was set to 100 °C. Throughout the process, the mixture was carefully observed as it transitioned from phase separation to a milky white state and eventually became transparent. Once the mixture reached a transparent state, heating was maintained for an additional 10 min and then turning off the heater. While the reaction mixture was still warm and relatively fluid, the magnetic stir bar was removed. Finally, after cooling, a colorless and transparent viscous substance was obtained. The product’s chemical structure was determined through ^1^H NMR and ATR-FTIR analyses.

### Masked Stereolithography Printing

HEMA and DPBMA were mixed at a weight ratio of 1:4 and stirred magnetically at 100 rpm until a uniform mixture was obtained. Subsequently, 0.5 wt % of the photoinitiator (BAPO) was added to produce the liquid printing ink. Based on functional requirements, a photochromic additive was incorporated into the previously prepared liquid printing ink. To minimize bubble formation during stirring, a magnetic stirrer was used at a controlled speed of 50 rpm to ensure thorough mixing. The specific photochromic additives included 1-(2-hydroxyethyl)-3,3-dimethylindoline-6′-nitrobenzopyran (SP1, 0.02 wt %) and (E)-3-(2-(2-hydroxyphenylethenyl)-5-methoxy-3,3-dimethyl-3H-indol-1-ium-1-yl)­propane-1-sulfonate (SP2, 0.02 wt %). The prepared spiropyran additive (SP1 or SP2) was incorporated into the printable ink for future use. The detailed ink formulations are reported in Table S1. Printing was conducted via a bottom-up 3D printer (Phrozen Sonic 4K 2022) with a 405 nm ParaLED Matrix 3.0 light source with 2.5 mW/cm^2^ irradiance. The print models were prepared via a Phrozen Dental Synergy Slicer, and all the samples were printed with a layer height of 100 μm and an exposure time of 5.5 s. Following the printing process, the printed samples were cleansed via a paper towel moistened with isopropyl alcohol to eliminate residual resin at the surface. Subsequently, the samples were air-dried for 2 min, and then UV postcured (405 nm, LED radiant of 6 W) for 10 min at ambient temperature.

### Preparation the University Logo badge

To fabricate an environmentally responsive badge, PR1 and PR2 were used to print a university logo that exhibits UV light and thermal responsiveness, respectively. The printed logos from PR1 and PR2 were positioned within a 25 mm circular mold. Subsequently, a nonphotochromic ink (PR3) was poured over the logo to encapsulate it and then cured under 405 nm UV light to finalize the sealing process. After cured, the badge was demolded to obtain the final responsive badge.

### Characterizations

^1^H NMR spectroscopy of DPBMA and its synthesis precursor was performed using nuclear magnetic resonance (NMR) on a 400 MHz Varian VXR spectrometer, with the sample dissolved in DMSO-*d*_6_ at 25 °C and recorded over 16 scans.

Attenuated total reflection-Fourier transform infrared (ATR-FTIR) spectra were acquired via a Bruker VERTEX 70 spectrometer equipped with a diamond single-reflection ATR accessory and scanned over the range of 4000--500 cm^–1^. To further investigate the changes in the absorbance of the dynamic ester, an ATR accessory with a heater was used to record the spectra across a temperature range of 40 to 160 °C.

The thermal stability of the cured resins was analyzed via thermogravimetric analysis (TGA) on a TA Discovery Instruments Q5500 instrument with an autosampler using a temperature ramp from 25 to 700 °C at a rate of 10 °C min^–1^ under atmospheric flow.

Tensile testing was performed on an Instron testing apparatus equipped with a load cell capacity of 1 kN to ascertain the mechanical characteristics of the materials under examination. The applied strain rate was 5 mm/min, and the samples for tensile testing were printed in a dumbbell configuration with dimensions of 48.75 × 3.25 × 1.3 mm^3^. The measurement was repeated five times to ensure reproducibility.

The glass transition temperature (*T*_g_) was measured via a TA Instruments Discovery HR20 instrument in DMA mode (1 Hz, 0.2% strain, heating rate: 3 °C min^–1^). The test samples had dimensions of 20 mm × 5 mm × 2 mm.

A UV–vis-NIR spectrophotometer (Lambda950, PerkinElmer) was used to measure the reflectance and transmittance of the photochromic film across a wavelength range of 190–2500 nm, featuring a dual-beam configuration and an integrating sphere. The International Commission on Illumination (CIE) diagram and the coordinate was obtained using the transmittance data from UV–vis-NIR spectrum.

UV–vis spectra of the photochromic compounds were recorded on a Hewlett-Packard HP 8543 spectrometer in a quartz cuvette with a path length of 1 cm. Irradiation was performed in situ via an optic fiber-coupled LED (Thorlabs) (455 nm, 1 A; 365 nm, 1 A). Solutions in acetonitrile or Milli-Q water were prepared the day before the measurements were taken and stored in the dark. The final concentration of the solutions was 2 × 10^–5^ M.

A portable 365 nm UV flashlight (5 W) and LED visible light (5 W) were used to excite the photochromic compounds and induce a color change process. Additionally, the heat gun was set to 80 °C and used to induce color change and shape reprogramming.

The reshaping of the printed specimen was performed by heating it to 80 °C using a heat gun. At this temperature, the material becomes highly malleable. External manual force was applied to bend, twist, or fold the sample into a temporary shape. Once the desired shape was achieved, the heat gun was removed while maintaining the external force. Once cooled, the printed specimens retained its programmed shape. This reshaping process is repeatable, allowing the material to be reprogrammed into various shape.

## Results and Discussion

Adaptive materials, which integrate real-time responsiveness and multifunctionality through a dynamic interplay between structure and function, represent a significant advancement in smart fabrication. Among additive manufacturing, mSLA printing offers a scalable photopolymerization process that provides a promising platform for adaptive manufacturing when functional additives are incorporated. As illustrated in Figure 2, the introduction of dynamic covalent networks (DCNs) enables the printed materials with programmable shape memory and reprocessability. In parallel, the incorporation of spiropyran additives imparts stimuli-responsive color changes. The combination of these functionalities endows 3D-printed constructs with adaptive behavior in response to external stimuli. These capabilities directly address the limitations of conventional mSLA printing materials, which often lack tunability and postfabrication adaptability. Specifically, the incorporation of dual dynamic covalent bonds (DCBs) facilitates bond exchange and topological rearrangement under elevated conditions, enabling shape reprogramming and recovery without compromising structural integrity. Furthermore, the photochromic nature of spiropyran provides a visual feedback mechanism, allowing printed components to dynamically respond to light and heat, thereby extending their utility beyond static applications. This advancement establishes mSLA printing as a viable strategy for adaptive manufacturing, where the properties and functionalities of printed materials can evolve in response to environmental stimuli.

**Figure 2:**
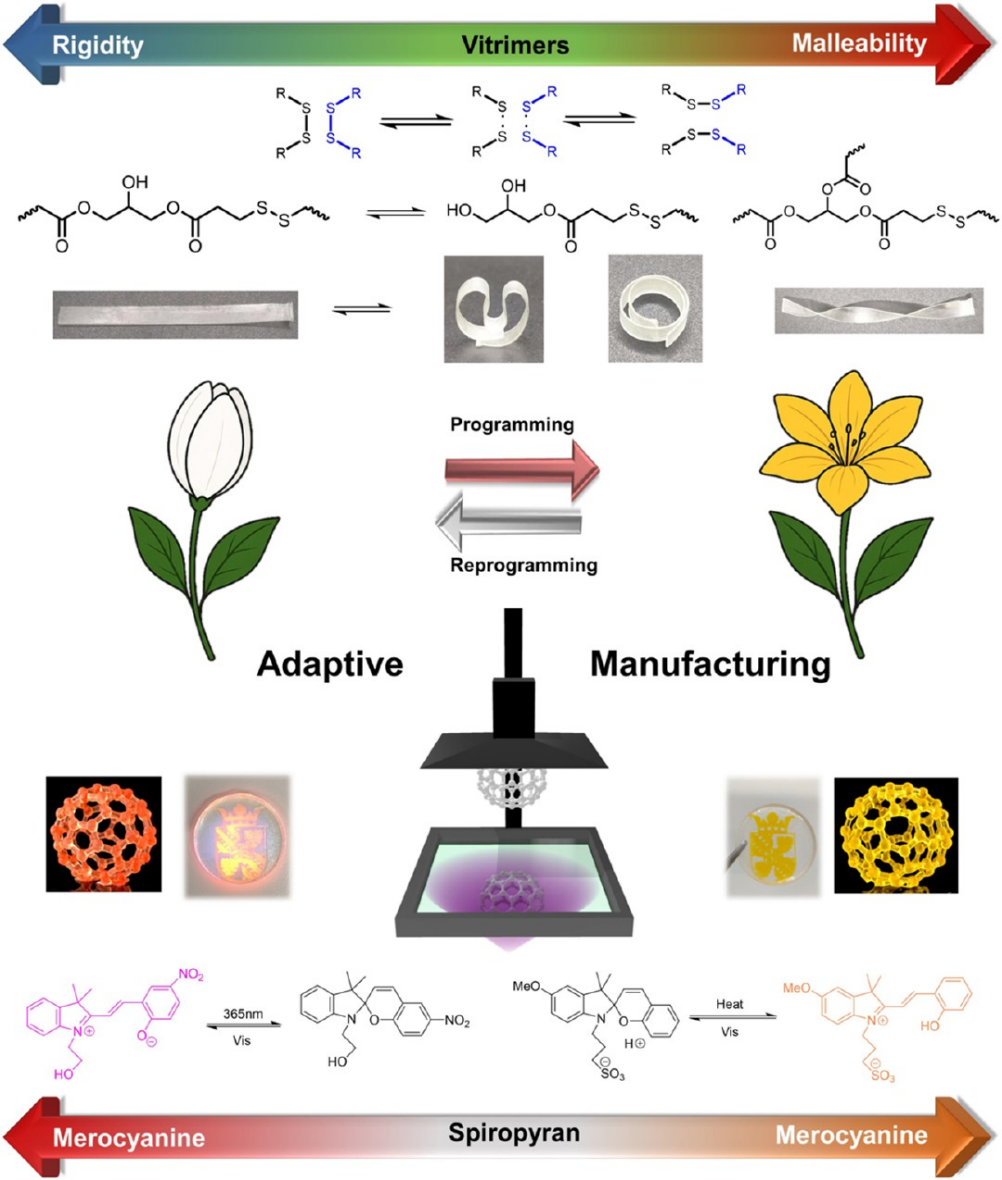
Illustration for the adaptive manufacturing.

The printing process was successful for all formulations, yielding stable prints, with specific formulations detailed in Table S1. The thermal stability and thermally activated shape reconfigurability of the printed samples were investigated via TGA and ATR-FTIR equipped with a heater. First, the heat resistance of the molecular switches (SP1 and SP2) was confirmed, showing only a 4–5% weight loss before reaching 200 °C (Figures S4 and S5). To further assess the thermal stability of the switchable prints, a series of thin films were printed using the aforementioned resin formulations. A series of thin films of PR1 (containing the photochromic switch SP1), the PR2 (containing the thermochromic switch SP2), and the PR3 film (without molecular switches) were analyzed. As shown in Figure 3a–c, the comparative analysis indicated that their thermogravimetric degradation curves exhibit very similar trends, with a mass loss of less than 3% occurring at approximately 100 to 120 °C, mainly due to the hygroscopic water loss. Additionally, the incorporation of photochromic or thermochromic additives slightly decreased the temperature at which 5% weight loss occurred from 250 to 230 °C (Figures 3a-c). To better understand the behavior of the material during heating and cooling, infrared spectroscopy was performed over a temperature range of 40 to 160 °C (Figure 3d). The results revealed that as the temperature increased, the infrared spectrum exhibited a redshift in the absorption band at approximately 3500 cm^–1^, indicating a weakening of hydrogen bonding within the polymer chain network.^32^ This weakening of intermolecular forces facilitates shape reprogramming at elevated temperatures by softening the material’s mechanical behavior. Conversely, the absorption shifted back to its original state as the temperature decreased, indicating the reversible nature of the hydrogen bonding interactions.

**Figure 3:**
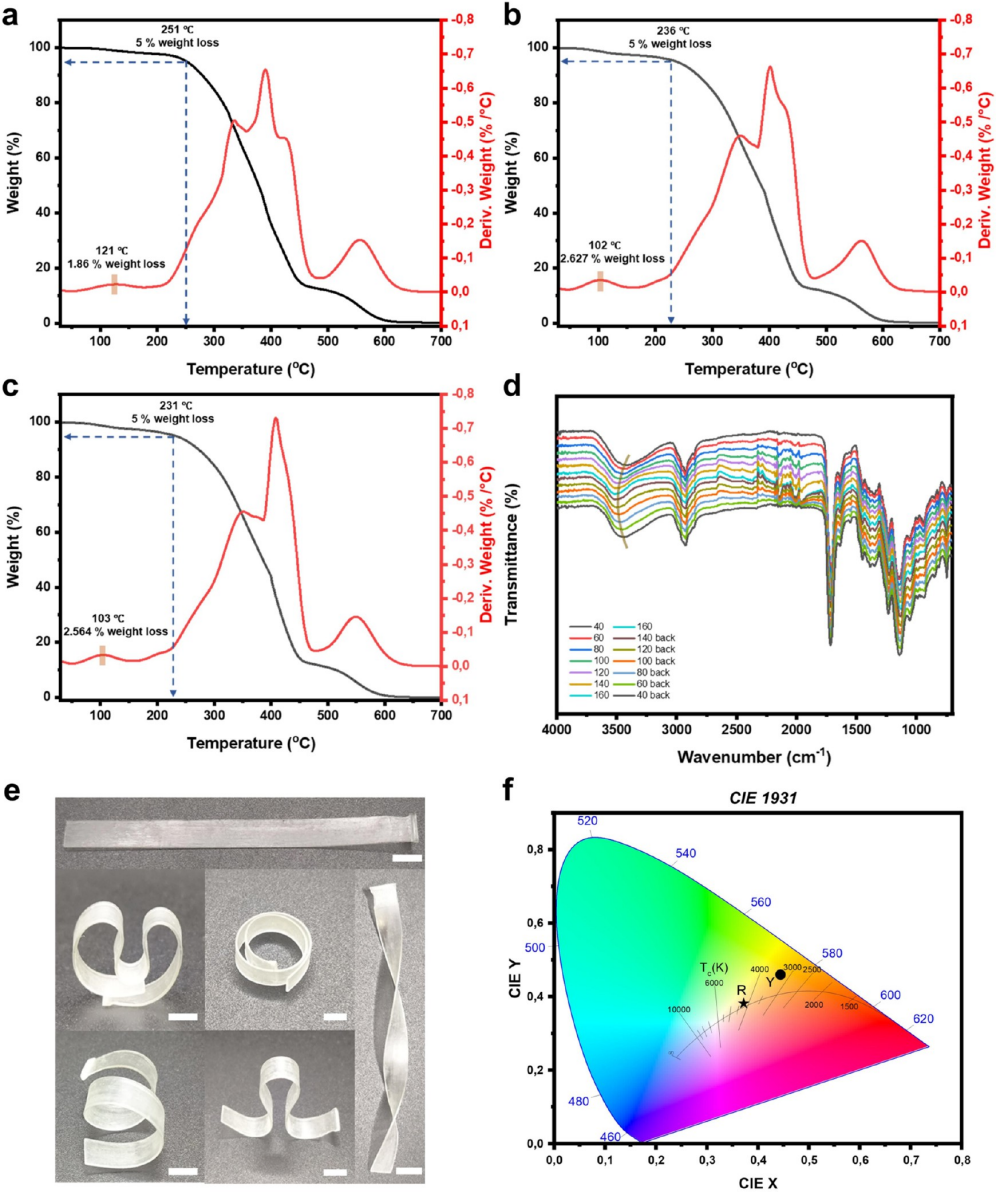
TGA curves of the printed films of (a) PR3, (b) PR1 and (c) PR2. (d) ATR-FTIR spectra of the PR1 film in the temperature range of 40 to 160 °C. (e) Visual representation of shape reconfiguration of printed spline (Scale bar = 6 mm). (f) CIE 1931 chromaticity diagram for the emission of films with SP1 or SP2. “R” represents the emission of the PR1 film, and “Y” represents the emission of the PR2 film.

The printed objects (Figure 3e) demonstrated thermal shape reprogramming, evidenced by the reshaping of the thin printed splines. Benefiting from the incorporated dual dynamic networks, the material exhibits both toughness (Figure S6) and a moderate glass transition temperature (80 °C, *T*_g_) (Figure S7). The International Commission on Illumination (CIE) diagram is used to represent the color of the photo- and thermally responsive films (PR1 and PR2) after exposure to UV light or heat. For photochromic materials, high transmittance is essential as it allows effective light penetration, enhancing the material’s responsiveness to light stimuli and maximizing the visibility and contrast of photoinduced color changes. The emission of the two types of photochromic polymeric films, based on PR1 and PR2 respectively, is presented on the CIE chromaticity diagram. The emission color coordinates for the PR1 film (referred to as R in Figure 3f) are 0.372 and 0.381, and for the PR2 film (named as Y in Figure 3f), the coordinates are 0.444 and 0.459.

In this study, two types of spiropyran-based molecules were separately incorporated into a photocurable resin to develop photo- and thermally responsive printed structures. Photoswitch SP1 was incorporated into the resin PR1, the University logo was printed, and the UV-responsive behavior of the printed logo was tested. Upon exposure to 365 nm UV light, the logo became clearly visible, demonstrating a color change to red (Figure 4b). The color change results from the reversible transition between the closed spiropyran (SP) form and the open merocyanine (MC) form. In the closed SP state, the molecule adopts a nonconjugated structure, lacking an absorbance peak in the visible spectrum, rendering it colorless. However, upon exposure to UV light, the molecule undergoes ring-opening to form the planar, conjugated MC state, which absorbs visible light and results in a distinct color change (Figure 4a). The fluorescent mechanism of spiropyran is primarily driven by its isomerization under ultraviolet (UV) light. Upon UV exposure, the chromophore, typically a benzene ring, absorbs light energy, transitioning from the ground state to the excited state. As the molecule returns to the ground state, energy is released in the form of photons, resulting in fluorescence.^33^ The whole process of pattern emergence and luminescence is provided in Video S1. As shown in Figure 4c–d, the SP-to-MC transition was completed in approximately 250 s, indicating a significant color change in a relatively short period. This rapid response time highlights the system’s capability to undergo visible transitions under 365 nm UV stimulation. Furthermore, the printed complex hollow fullerene structures provided a clearer visual representation of the color fading process (MC-to-SP transition) in a 3D model (Video S2). The digital images in Figure 4f–g vividly illustrate the high-resolution and color-retention properties of the film. Notably, the pink color required significantly longer time (approximately 3000 s, as shown in Figure 4g) to revert to its original state, indicating potential applications in areas requiring extended color retention, such as smart windows, where durability and accuracy are crucial. This photochromic film, when applied to windows, offers a promising approach for enhancing building energy efficiency by dynamically adjusting light transmission to reduce indoor heating and cooling demands. This functionality indirectly supports carbon neutrality efforts and actions against climate change by lowering the energy consumption associated with air conditioning systems.^25^

**Figure 4:**
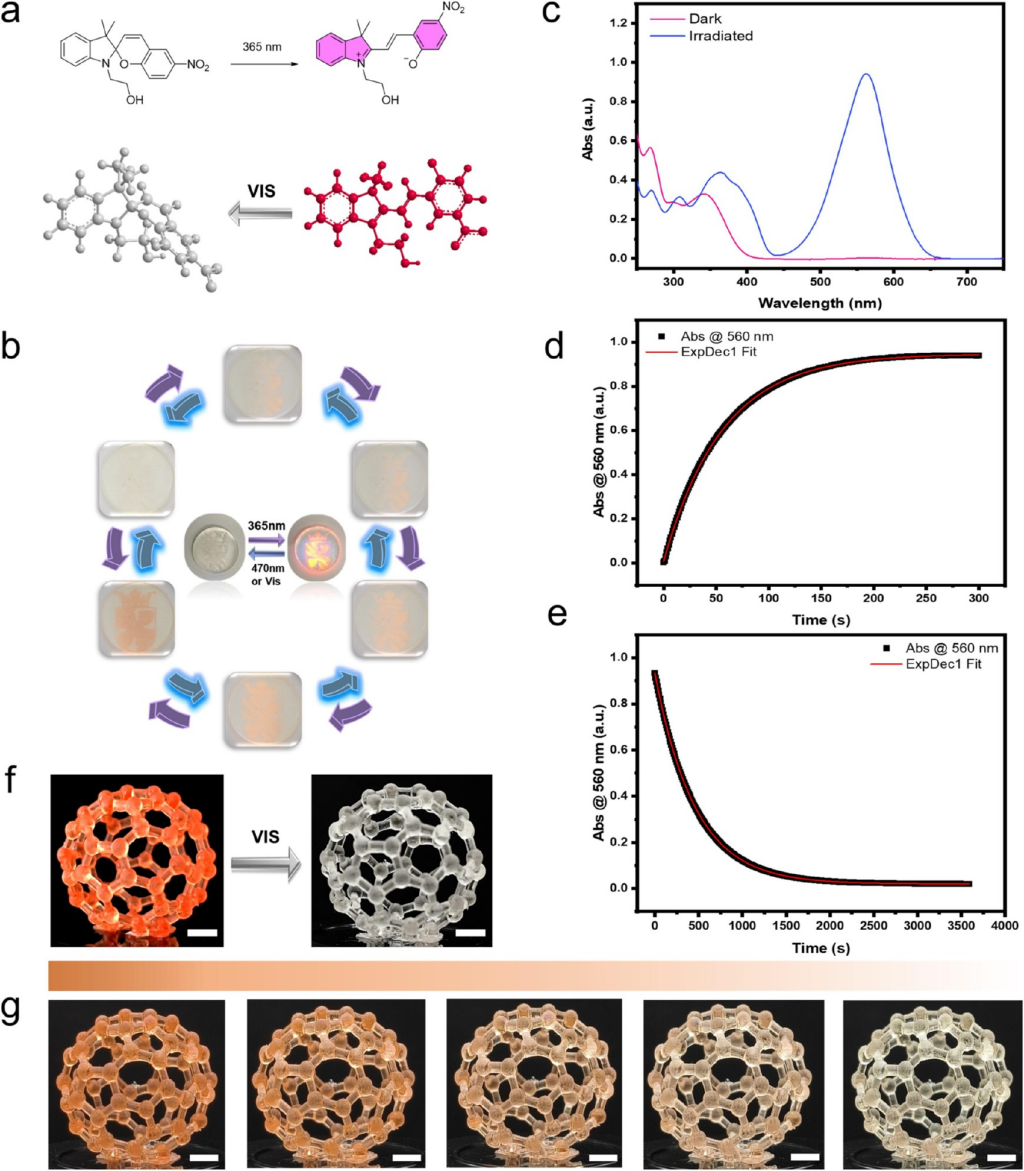
(a) SP-to-MC light induced isomerization for SP1. (b) UV response color change cycle of the university logo (diameter= 25 mm). (c) UV–vis absorption spectra of SP1 in acetonitrile before (purple) and after 365 nm UV exposure (blue). (d) Time evolution of the UV–vis absorbance intensity at 560 nm. (e) 3D structural MC–to-SP transition of SP1 under LED light. (f) 3D-printed photochromic fullerene structures. (g) Time evolution of model fading under LED light over 10 min. The five images, from left to right, show the color changes at intervals of 0, 31, 69, 113, and 600 s under continuous light irradiation (scale bar, 5 mm).

Classically, spiropyrans absorb energy from UV radiation to achieve molecular switching. More recently, a series of protonated merocyanine has been developed, which are able to ring-close, to form their corresponding SP form upon irradiation with visible light. The reverse process spontaneously happens in the dark to yield the protonated merocyanine form.^31,34^ Moreover, the ring opening reaction is associated with the appearance of coloration, thanks to the increase in conjugation of the molecule. The ring-opening process is thermodynamic and is accelerated by the application of heat. In this study, we utilized the spiropyran (SP2), generated upon irradiation with visible light, as a component in a printable ink formulation where the color change can be stimulated by temperature. Using the same protocol previously discussed, both the university logo and the fullerene model were prepared with resin PR2. The mechanism underlying the reversible transition of SP2 is analogous to that of SP1, involving a ring-opening or ring-closing process (Figure 5a). As shown in Figure 5b and Video S3, the logo exhibited no fluorescence, and the color change occurred at a moderate rate (Figure 5d). The time evolution of the UV–visible absorbance (Figure 5c) in the visible wavelength range for the SP–to-MC and MC–to-SP transitions is presented in Figures S12 and S13. The entire color fading process of the 3D model is provided in Video S4. Notably, the color change takes approximately 2500 s (Figure 5d) to reach full contrast, whereas the yellow color under visible light faded within just 25 s (Figure 5e), indicating that light-generated SP2 bears a stable temperature response suitable for temperature indicators, with potential applications in memory retention and enhanced accuracy.^35^

**Figure 5:**
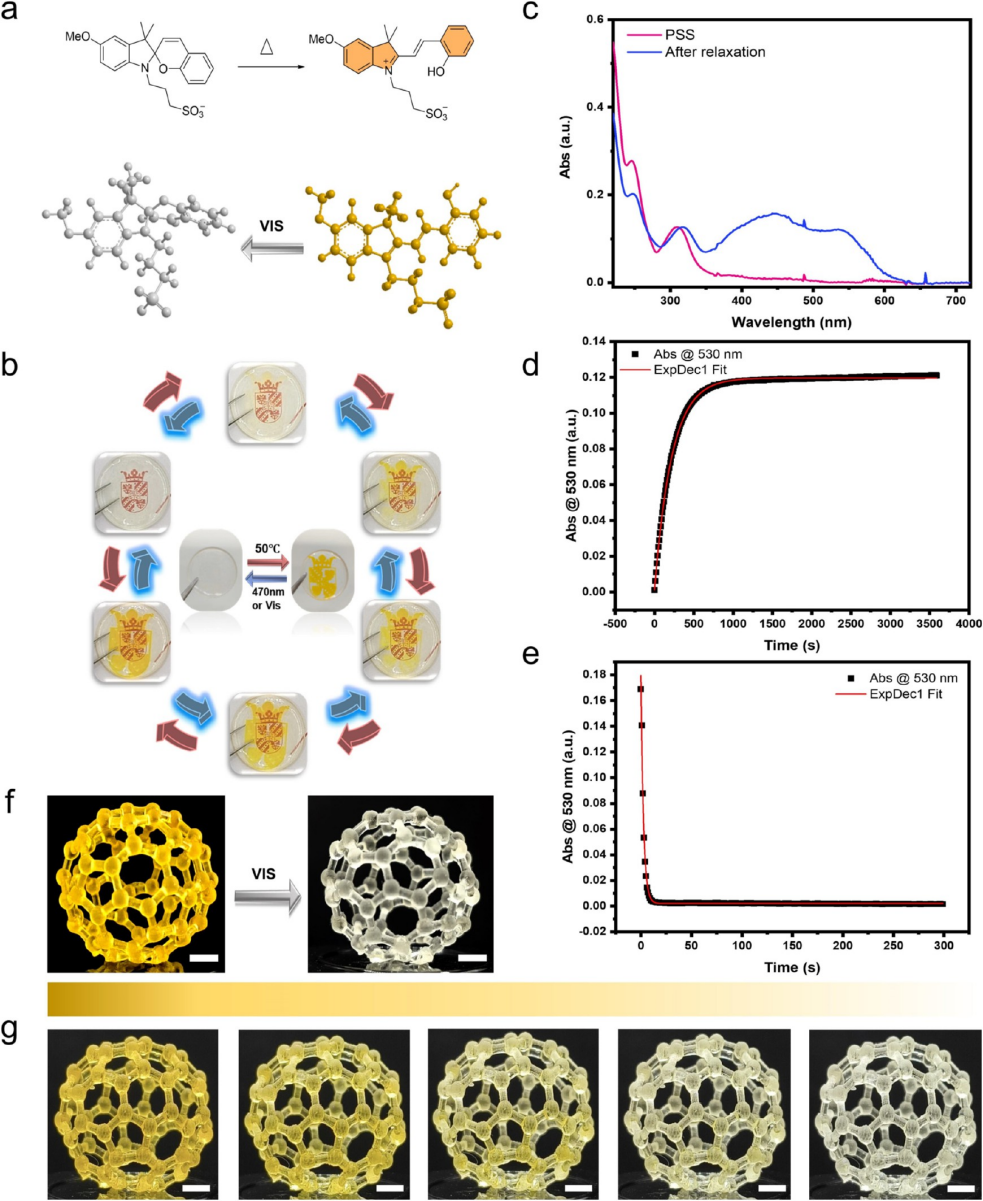
(a) Thermal isomerization from SP2 to its more thermodynamically stable open protonated merocyanine form. (b) Thermal response color change cycle for the university logo, diameter: 25 mm. (c) UV–vis absorption spectra of SP2 in water before and after heat. (d) Time evolution of the UV–vis absorbance intensity at 530 nm. (e) Structural MC–SP transition of SP2 under LED light. (f) 3D-printed photochromic fullerene structures. (g) Time evolution of model fading under LED light over 6 min. The five images, from left to right, show the color changes at intervals of 0, 24, 43, 67, and 360 s under continuous light irradiation (scale bar = 5 mm).

Thermoplastic 3D objects prepared from extrusion printing exhibit lower temperature resistance than thermosetting 3D printing materials via ink curing, mainly because thermoplastics are linear polymers that can melt and flow instead of being permanently fixed via chemically cross-linked networks.^36^ Vitrimer printing altered this phenomenon, as the dynamic covalent bonds reprogrammed the printed structures and prevented geometric collapse through thermally activated DCNs. As previously reported by Chen and co-workers, dual dynamic vitrimers that contain disulfide and ester bonds can be reprocessed when heated at elevated temperatures.^17^ In the polymer network formed by mSLA printing, the stable and irreversible C–C bonds resulting from methacrylate photopolymerization constitute the fixed phase, which retains the original shape. In contrast, disulfide bonds and β-hydroxy esters form the reversible phase, allowing chain segment mobility when the temperature exceeds the bond exchange threshold. As shown in Figure 6a–f, upon heating the material to its *T*_g_, the reversible phase softens, enabling chain mobility and polymer deformation under external force. After maintaining the deformed shape, lowering the temperature reduces the segment mobility, halts bond exchange, and stabilizes the temporarily reprogramming shape. When reheated, the reversible phase reactivates, allowing chain segments to move again. Driven by internal stress, the polymer returns to its initial shape, completing the shape memory cycle. In this work, incorporating thermally responsive molecules into the photosensitive resin endowed the material with dual responsiveness, allowing it to adapt both its shape and color in response to temperature changes. By mixing SP2 with the resin PR2, we successfully printed advanced structures, such as flowers, that demonstrated simultaneous shape and color changes, showcasing their potential as adaptive 3D-printed materials. Under heat flow at 80 °C, the flower transitions from a closed to an open state while its color shifts from colorless to yellow, demonstrating both shape transformation and chromatic response (Figure 6g–j, Video S5). This process is fully reversible, with the flower returning to a closed state as the color fades upon cooling (Figure 6k–n, Video S6). These dual-responsive materials offer customizable geometries and color transitions, making them highly adaptable for temperature-triggered applications such as 4D printed bionic smart materials^37^ and environment and performance indicators.^38^

**Figure 6:**
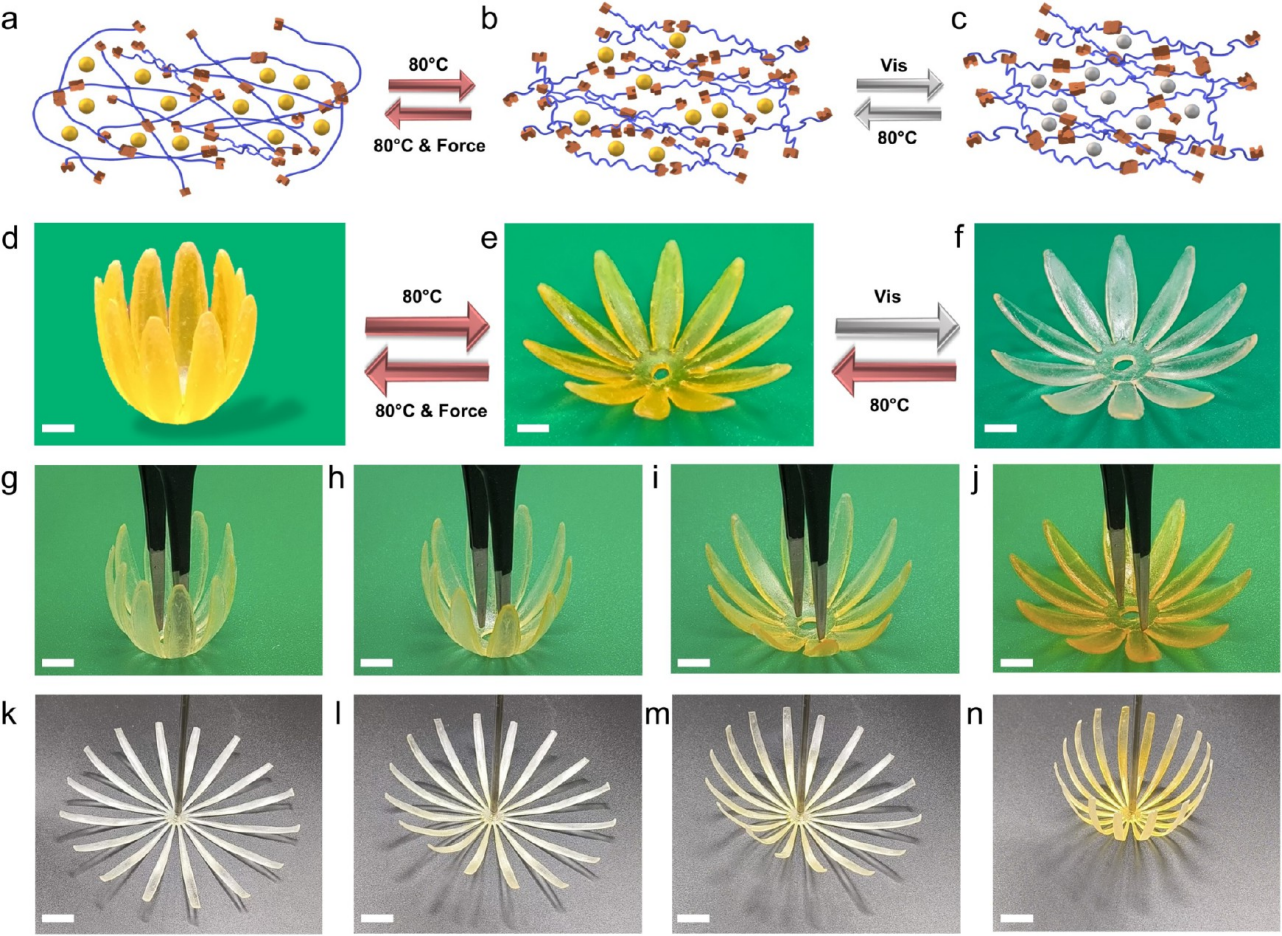
(a–c) Schematic representation of network topological isomerization and thermochromic transition within the 3D printed structures. Blue lines indicate polymer chains, yellow dots represent SP2 molecular switches, and squares (either attached or detached) represent dynamic covalent bonds (DCBs) in the polymer network. (d–f) Overview of the transition from open to closed and from colorless to yellow; scale bar, 5 mm. (g–j) Printed flower bloom at 80 °C and color transition process; scale bar, 5 mm. **(k-n)** Printed flower bloom at 80 °C and color transition process; scale bar, 6 mm.

## Conclusions

In summary, we present the development of environmentally friendly, photochromic and dual-responsive thermochromic printing inks through a solvent-free polymerization process. By incorporating dynamic covalent bonds and spiropyran-based molecular switches, the resulting printed structures demonstrate remarkable shape memory properties, high thermal stability, and tunable chromatic responses. The dual-responsive behavior, where both shape and color transitions are activated thermally, paves the way for advanced applications in domains such as anticounterfeiting, optoelectronics, and information encryption. The use of dynamic covalent networks in the printing process allows for highly customizable material properties that can endure thermal reconfigurations without geometric deformation. This work significantly advances the design of sustainable 3D printing ink that aligns with both cost-effective and functional demands, laying the groundwork for future innovations in smart material applications.

## Supplementary Material















## Notes

^1^H NMR spectra of DPBMA and SP2, ATR-FTIR spectra and TGA analysis of spiropyran, glass transition temperature (*T*_g_), tensile strain measurements, UV–visible spectra for SP1 and SP2, transmittance and reflectance curves for PR1 and PR2 film, table for the resin formulations, tables of fitted data for spiropyran UV–Vis absorption parameters and the digital prototype utilized for printing (PDF)
Molecule returns to the groundstate, energy is released in the form of photons (MP4)
Visual representation of the color fading process (MC-to-SP transition of SP1) in a 3D model (MP4)
Thermal response color change cycle for the university logo (MP4)
Color fading process (MC-to-SP transition of SP2) of the 3D model (MP4)
Flower transitions from a closed to an open state while its color shifts from colorless to yellow (MP4)
Flower returning to a closed state along with a color shift from colorless to yellow (MP4)
