# Supplementary material for: 3D-Printed Dual Photo- and Thermally Responsive Materials for Smart Adaptability
Source: Macromolecules. 2025 Jul 15;58(15):8249–59. doi: 10.1021/acs.macromol.5c00567 (PMC12356063; doi:10.1021/acs.macromol.5c00567)
Supplement: Supplementary file 1 [file ma5c00567_si_001.pdf]

3D Printed Dual Photo- and Thermally Responsive Materials for Smart Adaptability

Tao Zhang[1,2], Gianni Pacella[1], Kunlin Chen[1,3], Ting Ye[1,3], Giuseppe Portale[1], Vincent S. D. Voet[2], Rudy Folkersma[2] and Katja Loos[1]

[1]Macromolecular Chemistry and New Polymeric Materials, Zernike Institute for Advanced Materials, University of Groningen, Nijenborgh 3, Groningen 9747 AG, The Netherlands

[2]Circular Plastics, Academy Tech & Design, NHL Stenden University of Applied Sciences, Van Schaikweg 94, 7811KL Emmen, the Netherlands

[3]Key Laboratory of Eco-Textile, Ministry of Education, School of Textile Science and Engineering, Jiangnan University, Wuxi 214122, China

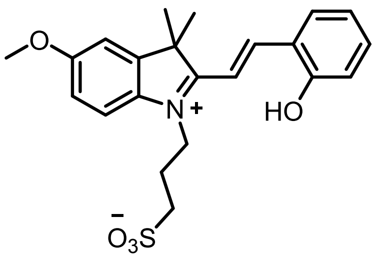

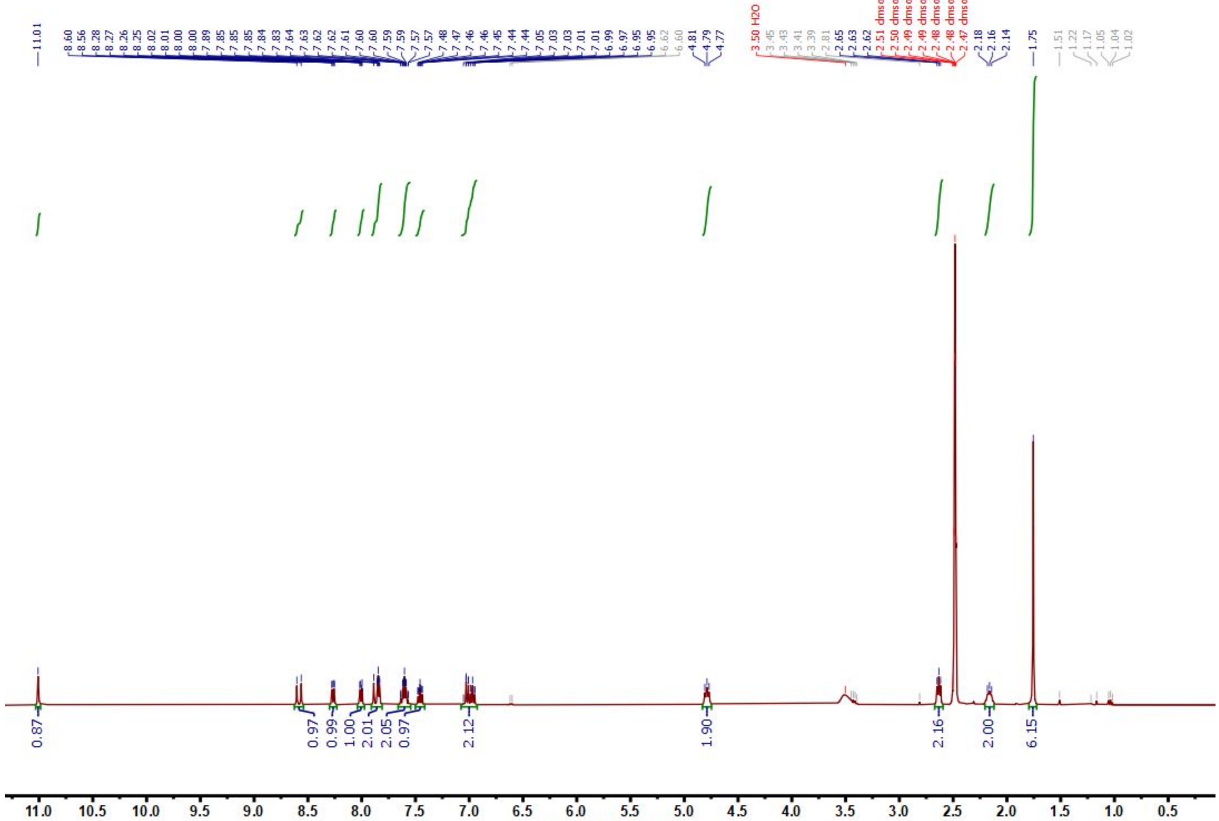

Figure S1. ^{1}H NMR spectra of SP2.

^{1}H NMR (400 MHz, dmso) δ 10.92 (s, 1H), 8.50 (d, *J* = 16.4 Hz, 1H), 8.24 (d, *J* = 7.9 Hz, 1H), 7.95 (d, *J* = 8.8 Hz, 1H), 7.81 (d, *J* = 16.4 Hz, 1H), 7.55 – 7.40 (m, 2H), 7.17 (dd, *J* = 8.9, 2.5 Hz, 1H), 7.07 – 6.93 (m, 2H), 4.77 (t, *J* = 8.0 Hz, 2H), 3.89 (s, 3H), 2.63 (t, *J* = 6.5 Hz, 2H), 2.16 (t, *J* = 7.6 Hz, 2H), 1.76 (s, 6H).

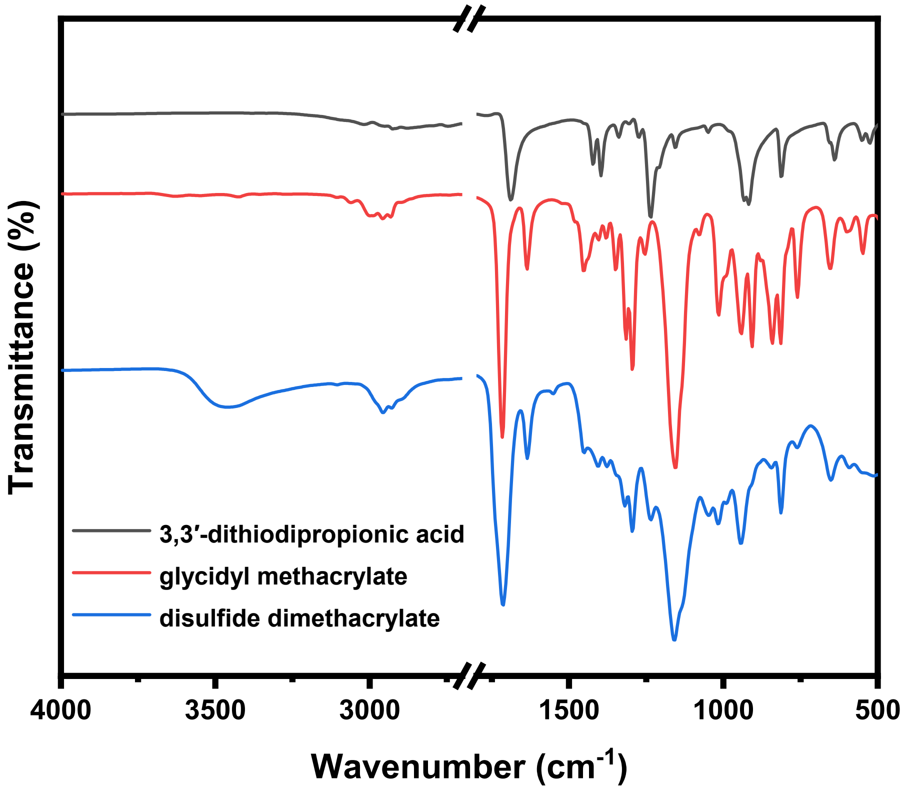

Figure S2. ATR-FTIR spectra of the precursors (3,3’-dithiodipropionic acid and glycidyl methacrylate) and DPBMA.

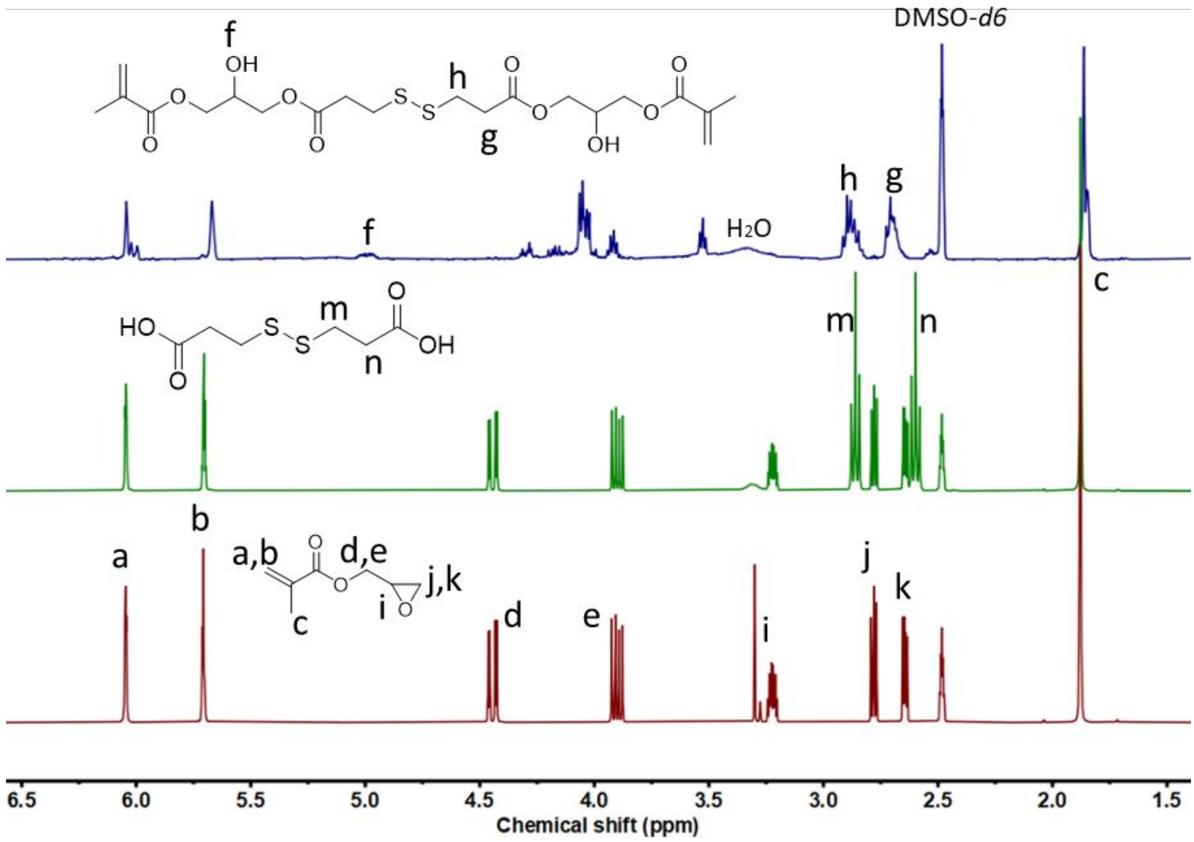

Figure S3. ^{1}H NMR spectra of glycidyl methacrylate (precursor), the mixture of (3,3'-dithiodipropionic acid and glycidyl methacrylate, and the synthesized DPBMA.

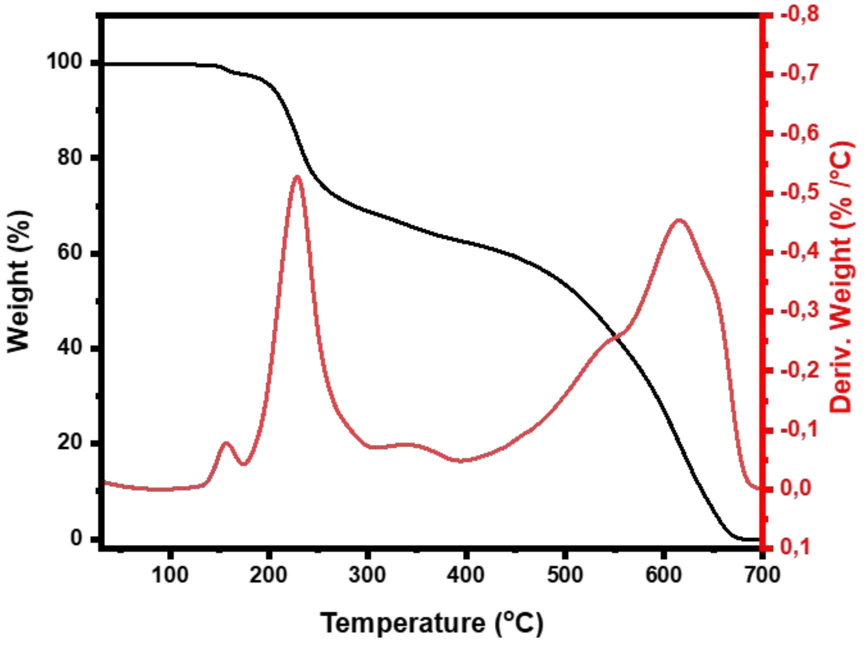

Figure S4. TGA and derivative thermogravimetric (DTG) curves of the printed polymer with SP1 (PR1 film).

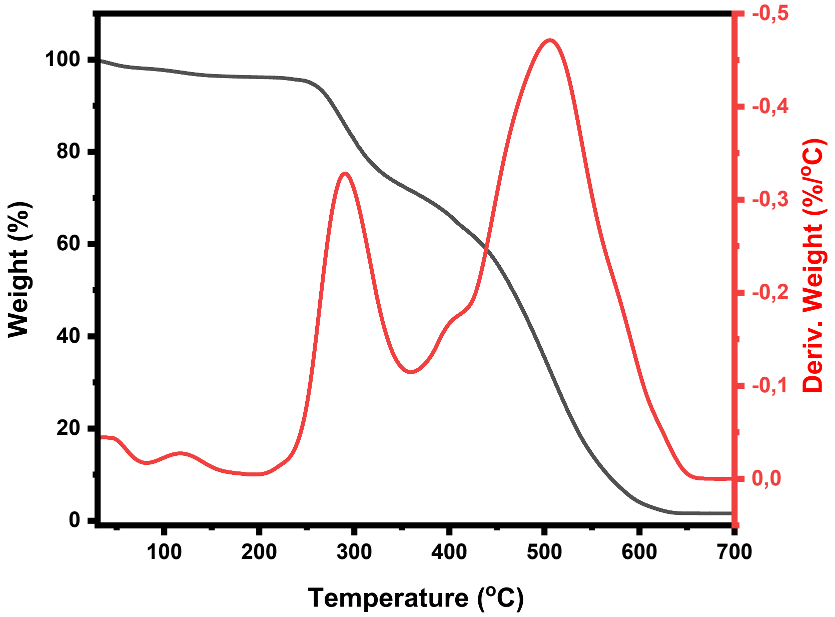

Figure S5. TGA and DTG curves of the printed polymer with SP2 (PR2 film).

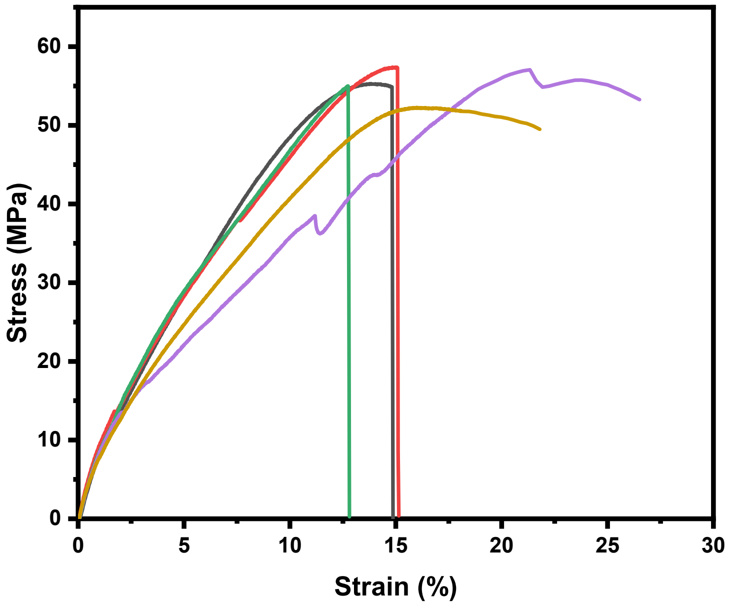

Figure S6. Stress–strain curves of the PR1 film (The measurement was repeated five times).

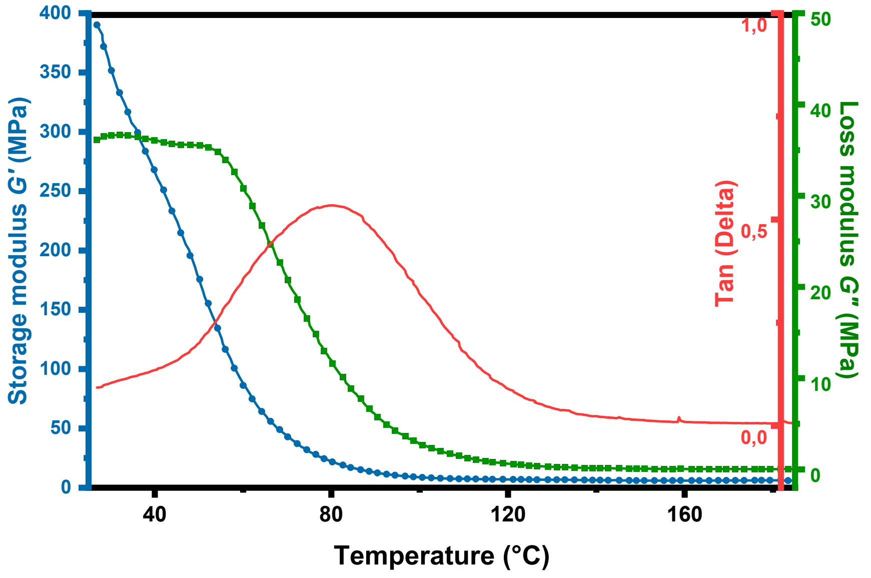

Figure S7. Storage modulus, Loss modulus, and Loss factor curves for PR3 film.

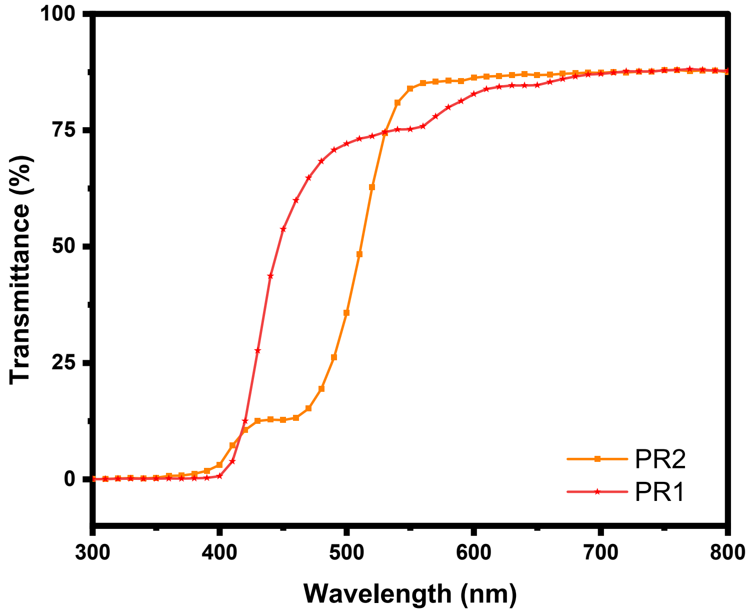

Figure S8. Transmittance curves of photochromic films with two spiropyrans (PR1 for red and PR2 for orange).

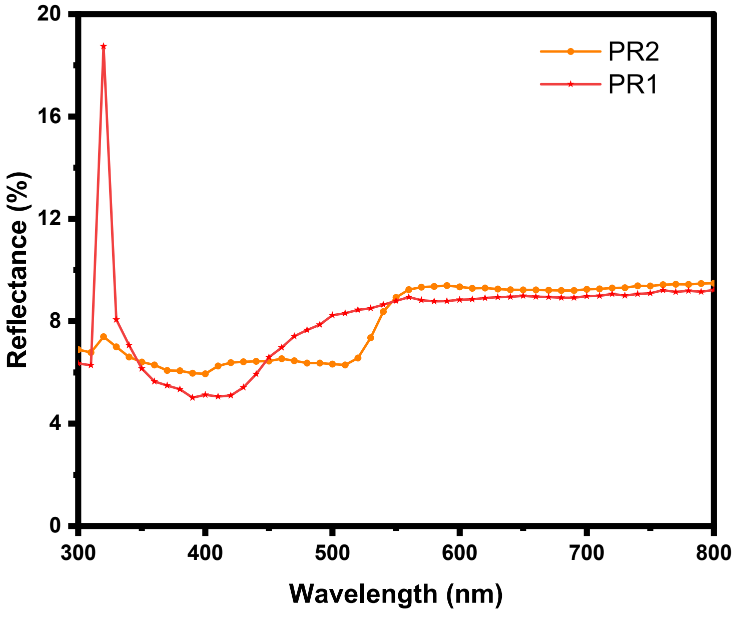

Figure S9. Reflectance curves of photochromic films with two spiropyrans (PR1 for red and PR2 for orange).

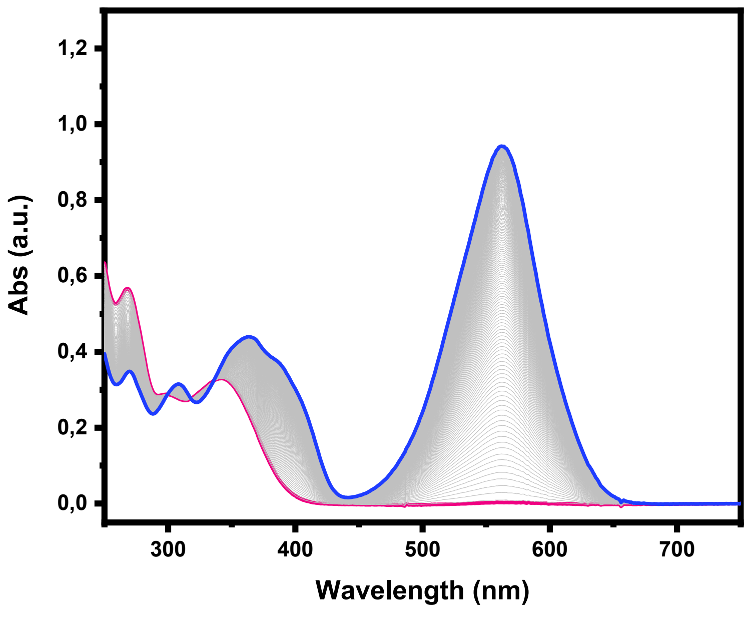

Figure S10. Time evolution of the UV–vis absorbance intensity for SP1 under 365 nm UV, Irradiation: 365 nm LED, 1A, over a duration of 0 to 300 s.

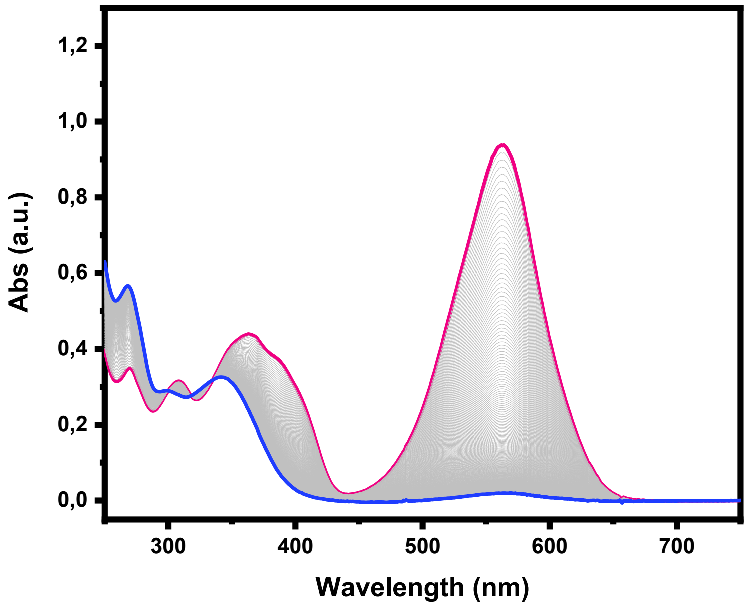

Figure S11. Time evolution of the UV–vis absorbance intensity for SP1 at the Photo Stationary State over a duration of 0 to 3590 s.

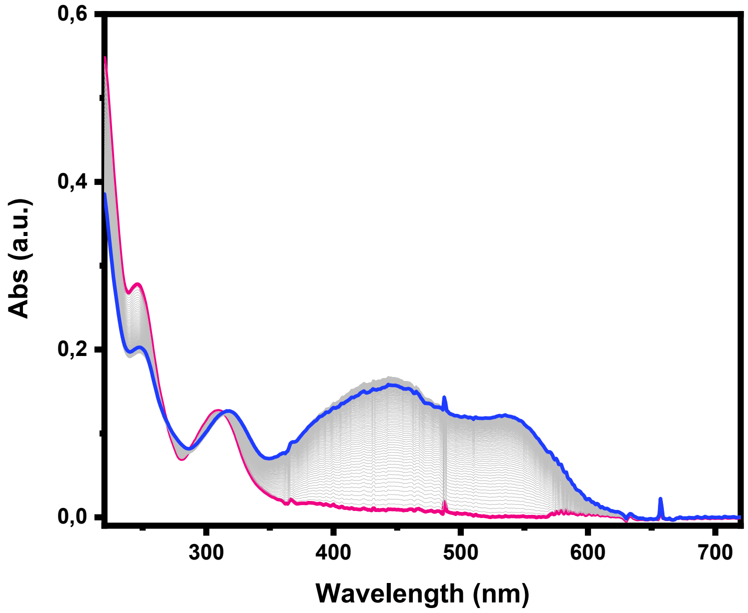

Figure S12. Time evolution of the UV–vis absorbance intensity for SP2 at 50 °C, over a duration of 0 to 3590 s.

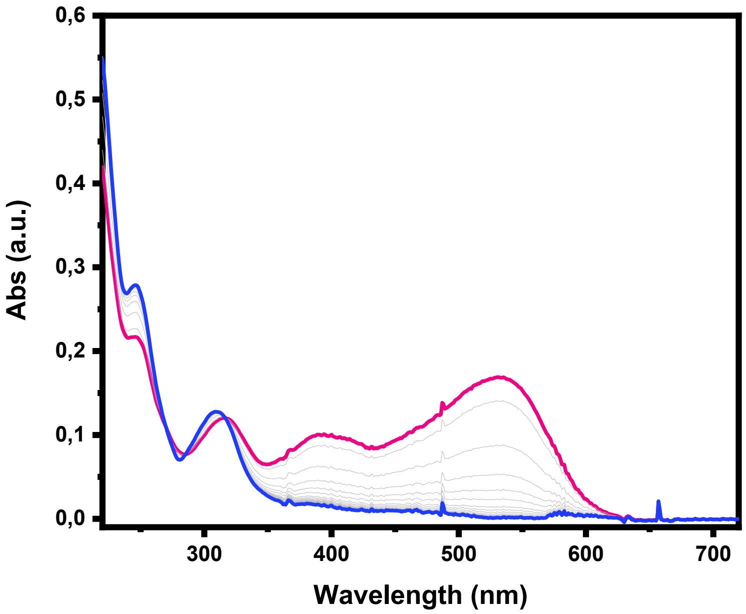

Figure S13. Time evolution of the UV–vis absorbance intensity for SP2 under Vis irradiation at 455 nm and 1A, over a duration of 0 to 300 s.

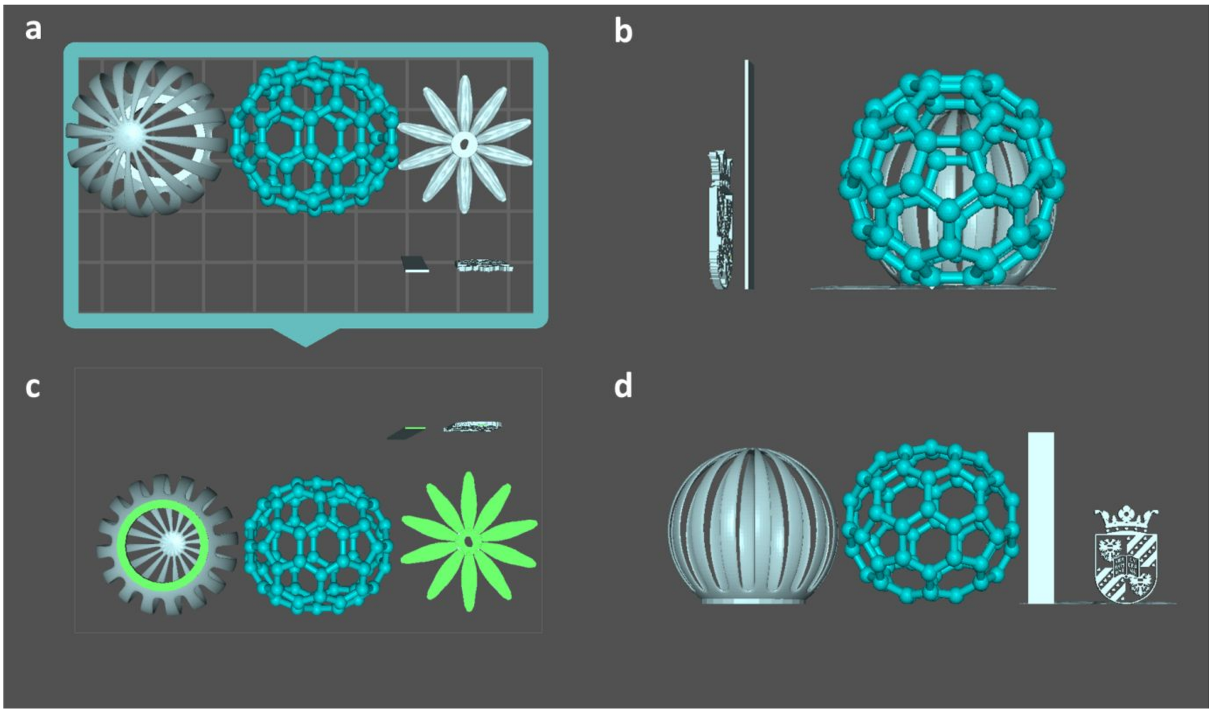

Figure S14. Schematic representations of the 3D-printed models from multiple perspectives: **(a)** top view, **(b)** right view, **(c)** bottom view, and **(d)** front view. (Models were downloaded from the Thingiverse website.)

Table S1. Composition of the evaluated resin formulations. Each formulation contains 0.5% w/w BAPO photoinitiator relative to the total resin weight. Specifically, PR2 contains 0.02% w/w SP1, while PR3 incorporates 0.02% w/w SP2.

Resin	Monomer (% w/w)			
	DPBMA	HEMA	SP1	SP2
PR1	80	20	Yes	No
PR2	80	20	No	Yes
PR3	80	20	No	No

Table S2. Fitted parameters for the SP1 absorbance increase at 560 nm over time under 365 nm UV irradiation, derived from UV-Vis absorption spectra.

Model	ExpDec1
Equation	y = A1*exp(-x/t1) + y0
Plot	Abs @ 560 nm
y0	0,94707 ± 2,90019E-4
A1	-0,93956 ± 7,25762E-4
t1	55,78546 ± 0,0893
Reduced Chi-Sqr	7,58579E-6
R-Square (COD)	0,99986
Adj. R-Square	0,99986

Table S3. Fitted parameters for the SP1 absorbance decrease at 560 nm over time, derived from UV-Vis absorption spectra.

Model	ExpDec1
Equation	y = A1*exp(-x/t1) + y0
Plot	Abs @ 560 nm
y0	0,01949 ± 1,4183E-5
A1	0,91433 ± 5,48543E-5
t1	456,95388 ± 0,04809
Reduced Chi-Sqr	3,58931E-8
R-Square (COD)	1
Adj. R-Square	1

Table S4. Fitted parameters for the SP2 absorbance increase at 530 nm over time under heat, derived from UV-Vis absorption spectra.

Model	ExpDec1
Equation	y = A1*exp(-x/t1) + y0
Plot	Abs @ 530 nm
y0	0,11977 ± 4,41443E-5
A1	-0,11849 ± 2,95741E-4
t1	221,35417 ± 0,86164
Reduced Chi-Sqr	5,28967E-7
R-Square (COD)	0,99868
Adj. R-Square	0,99867

Table S5. Fitted parameters for the SP2 absorbance decrease at 530 nm, derived from UV-Vis absorption spectra.

Model	ExpDec1
Equation	y = A1*exp(-x/t1) + y0
Plot	Abs @ 530 nm
y0	0,002 ± 8,26283E-5
A1	0,17751 ± 0,00125
t1	2,57635 ± 0,03102
Reduced Chi-Sqr	1,97699E-6
R-Square (COD)	0,98986
Adj. R-Square	0,9898
